# Characterising the plasma metabolome following high intensity interval exercise in continuous and intermittent hypoxia in overweight individuals

**DOI:** 10.1113/EP094252

**Published:** 2026-11-07

**Authors:** Jacky Soo, Maartje Cox, Paul Goods, Olivier Girard, Louise Deldicque, Monique Ryan, Philipp Nitschke, Nathan Lawler, Timothy Fairchild

**Affiliations:** ^1^ School of Allied Health (Exercise Science) Murdoch University Perth Australia; ^2^ Institute of Neuroscience Université Catholique de Louvain Louvain‐la‐Neuve Belgium; ^3^ The Centre for Computational and Systems Medicine (CCSM), Health Futures Institute Murdoch University Perth Australia; ^4^ The Centre for Health Aging, Health Futures Institute Murdoch University Perth Australia; ^5^ School of Human Sciences (Exercise and Sport Science) The University of Western Australia Crawley Western Australia Australia; ^6^ Australian National Phenome Centre, Health Futures Institute Murdoch University Perth Australia

**Keywords:** hypoxia, intermittent exercise, metabolism, metabolomics

## Abstract

The aim of this work was to examine changes in the plasma metabolome in response to high‐intensity interval exercise (HIIE) performed under three distinct hypoxia protocols. On separate days, 18 inactive individuals with overweight completed a HIIE protocol in randomised conditions: normoxia (N_Ex_‐N_Rec_), normobaric hypoxia (inspired O_2_ fraction ∼0.14) during both the work and recovery (H_Ex_‐H_Rec_), normobaric hypoxia during recovery only (N_Ex_‐H_Rec_) and normobaric hypoxia during work only (H_Ex_‐N_Rec_). Venous blood samples were collected before and after each HIIE for metabolite analyses by liquid chromatography–mass spectrometry and ^1^H‐nuclear magnetic resonance spectroscopy. Mean power output, averaged across the six work bouts, was lower in H_Ex_‐H_Rec_ compared to N_Ex_‐N_Rec_, N_Ex_‐H_Rec_ and H_Ex_‐N_Rec_ (all *P* ≤ 0.009), with no differences (all *P* ≥ 0.11) between the other conditions. Arterial oxygen saturation was lower in H_Ex_‐H_Rec_ compared to other conditions (all *P *< 0.001). Concentrations of pyruvate and closely associated metabolites (alanine, citrate, lactate) increased consistently across all conditions (all *P *< 0.001; Hedge's *g*: 0.43–1.73), without differences between conditions (all *P *> 0.37). The lactate to pyruvate ratio increased in all conditions, but did not differ between them. Free‐fatty acid (FFA) classes (saturated, monounsaturated and polyunsaturated FFAs) showed consistent increases (all *P *< 0.001), while monounsaturated and saturated triacylglycerol (TAG) classes significantly decreased following HIIE (both *P *< 0.04). There were no condition × time interaction effects for any metabolites. HIIE significantly altered the concentration of glycolytic and TCA cycle intermediates, whilst concomitantly increasing FFA and decreasing TAG concentrations. Although hypoxia applied either continuously throughout HIIE or intermittently (work or recovery) during HIIE altered exercise performance and oxygen saturation, it had limited additional effect on metabolite concentrations.

## Abstract (highlights)

- **What is the central question of this study?**
  Does addition of hypoxia amplify the acute metabolic response to high‐intensity interval exercise (HIIE) in previously inactive adults with overweight?
- **What is the main finding and its importance?**
  Hypoxia did not amplify the metabolic response to HIIE, but it may compensate for reduced mechanical work. Continuous hypoxia reduced power output yet produced a plasma metabolic response comparable to normoxia. Intermittent hypoxia preserved power output but did not amplify the metabolic response. Continuous hypoxia during HIIE generates similar systemic metabolic responses at lower mechanical loads, but whether these responses reflect intramuscular metabolism warrants further investigation.

## 1 INTRODUCTION

Physical inactivity and sedentary behaviours reduce skeletal muscle insulin sensitivity and oxidative capacity (Befroy et al., 2008; Rabøl et al., 2011), and impair metabolic flexibility (San‐Millán & Brooks, 2018). Exercise training protects against these detrimental metabolic effects, with higher volumes and/or intensities conferring greater metabolic benefits (Smith et al., 2023). High intensity interval exercise (HIIE) has gained traction as a potent stimulus for improving metabolic health (Coates et al., 2023). Characterised by brief bouts of high‐intensity efforts (≤4 min at ≥80% peak heart rate) interspersed with short recovery periods (Callahan et al., 2021), HIIE enables less trained individuals to reach intensities that stimulate skeletal muscle glycogen utilisation. Accordingly, HIIE is associated with improved metabolic outcomes in sedentary, overweight individuals (Kanaley et al., 2022; Whyte et al., 2010).

The acute metabolic effects of HIIE may be further enhanced by an additional physiological stressor such as hypoxia (Soo et al., 2023). Hypoxia favours a shift towards oxygen‐independent energetic pathways, including glycolysis and increased reliance on glycogen utilisation (Wadley et al., 2006), as indicated by elevated plasma lactate concentrations. However, exercising in hypoxia significantly reduces absolute exercise intensity due to lower arterial oxygenation (Billaut & Buchheit, 2013), which may blunt the overall exercise stimulus. To this end, studies have explored alternative hypoxia protocols that minimise performance decrements (Papoti et al., 2023; Roels et al., 2005). One such method is intermittent hypoxic exposure, where hypoxia is applied only during either the work or the recovery phase of HIIE (Dellavechia de Carvalho et al., 2023; Roels et al., 2005).

Given that increasing exercise intensity and hypoxic severity are associated with greater glycogen utilisation and reliance on glycolytic metabolism, blood lactate accumulation is commonly used as an indicator of metabolic perturbation, with most studies showing a significant increase in blood lactate during the initial phase of HIIE (Seiler & Sjursen, 2004). However, measuring blood lactate alone provides limited insight into the relative contributions of major energetic pathways, and may not fully capture the subtle influence of hypoxia on the interaction between aerobic and anaerobic processes.

Exercise and hypoxia exert profound and systemic effects on multiple pathways and intermediates independently, but their concerted effect on metabolites beyond lactate is not currently well described. A comprehensive analysis of metabolites in a biological system, termed metabolomics (Belhaj et al., 2021), has shed further light on the metabolic changes associated with exercise. For example, metabolomic studies adopting gas chromatography–mass spectrometry or liquid chromatography–mass spectrometry (LC‐MS) have detected distinct metabolic signatures associated with the modality and intensity of exercise (Danaher et al., 2015; Govus et al., 2025; Morville et al., 2020), and have also revealed changes in free fatty acid (FFA) and amino acid metabolism (Lou et al., 2014; Margolis et al., 2021) induced by hypoxia. Additionally, Zagatto et al. (2022) utilised nuclear magnetic resonance (NMR) spectroscopy to examine changes in the metabolic responses during high intensity exercise and showed that skeletal muscle metabolites associated with oxidation of long chain FFAs, TCA cycle metabolites and pyruvate metabolism (e.g., lactate, alanine, glutamate) were significantly altered. Given the diverse spectrum of metabolites that vary in mass, charge and polarity, a multiplatform approach will broaden the range of metabolites that can be assessed and may reveal a more complete understanding of the metabolic responses of hypoxia during HIIE in sedentary individuals with overweight.

Accordingly, the aim of this study is to utilise a multiplatform approach to examine the change in the plasma metabolome in response to HIIE performed under various hypoxia (two forms of intermittent hypoxia and one form of continuous hypoxia) conditions. Specifically, analyses targeted lipid oxidation as a key intermediate of aerobic metabolism and pyruvate metabolism which reflects the balance between aerobic and anaerobic metabolism. We hypothesised that HIIE would increase plasma FFA concentrations (reflecting reduced skeletal muscle uptake), the lactate to pyruvate ratio and pyruvate‐related intermediates. We further anticipated continuous hypoxia to produce the greatest increases in plasma FFA concentrations and lactate to pyruvate ratio.

## 2 METHODS

### 2.1 Ethical approval

Before the commencement of the study, participants were informed of the experimental procedures, as well as the possible benefits and risks, and provided written informed consent. This study was approved by the Human Research Ethics Committee of Murdoch University (2022/074) and performed in accordance with the *Declaration of Helsinki*, except for registration in a database.

### 2.2 Participants and inclusion criteria

Eighteen inactive (defined as fewer than two scheduled aerobic exercise sessions and <100 min of moderate to vigorous intensity aerobic exercise per week) and overweight (body mass index, BMI: ≥25.0 kg m^−2^) males (*n* = 13; 31 ± 8 years; BMI: 28.0 ± 3.0 kg m^−2^) and females (*n* = 5; 32 ± 10; BMI: 30.5 ± 3.6 kg m^−2^) volunteered to participate in this study.

Participants were excluded if they were smokers, had uncontrolled hypertension (blood pressure greater than 160/90 mmHg), had a prior history of heart disease, or had contraindications to exercise assessed using the Exercise and Sports Science Australia (ESSA) pre‐exercise screening tool. Additionally, individuals born and raised at altitudes exceeding 1500 m or who had travelled to an elevation exceeding 1000 m within 3 months prior to the study were excluded.

### 2.3 Experimental design

This study, conducted as part of a larger research investigation into exercise responses to perceptually regulated high‐intensity interval exercise under continuous and intermittent hypoxia, reports primary outcomes (metabolomics) not previously covered in analyses (Soo et al., 2025).

Participants visited the Exercise Physiology laboratory at Murdoch University on five separate occasions, comprising one familiarisation session and four main experimental trials. All experimental sessions were conducted in a randomised, crossover and counterbalanced design (Latin‐square), with each session separated by at least 4–7 days and conducted at the same time of day.

**FIGURE 1:**
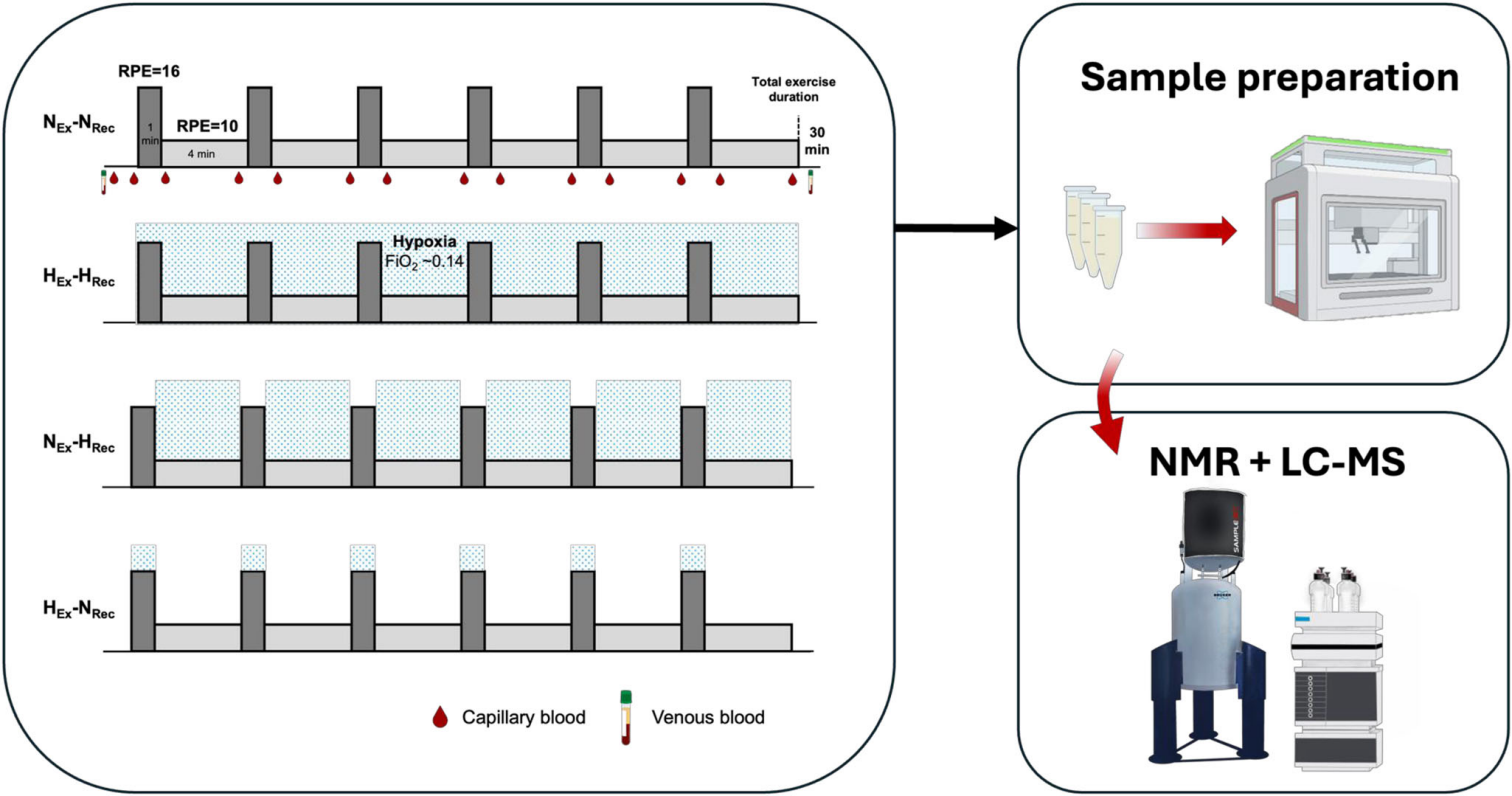
Experimental protocol. The high intensity interval exercise consisted of 6 × 1‐min work bouts, each interspersed with 4 min of recovery. The work and recovery bouts were performed at a perceptually regulated intensity corresponding to an RPE of 16 and 10, respectively. Participants completed four separate experimental sessions involving perceptually regulated high intensity exercise under the following conditions: (1) normoxic exercise/normoxic recovery (N_Ex_‐N_Rec_); (2) hypoxic exercise/hypoxic recovery (H_Ex_‐H_Rec_); (3) normoxic exercise/hypoxic recovery (N_Ex_‐H_Rec_); and (4) hypoxic exercise/normoxic recovery (H_Ex_‐N_Rec_). Peripheral oxygen saturation response was measured continuously throughout exercise. Capillary blood samples were taken from the fingertip and analysed for blood lactate levels at baseline (before any exercise), 1 min before the start of the HIIE protocol, ∼15 s after each work bout, and ∼45 s before the start of the next 1‐min bout. A venous blood sample was taken before and after exercise and immediately centrifuged. Blood plasma was transferred into microtubes for storage at −80°C until subsequent analysis. Metabolic profiling was performed on the plasma to quantify metabolites using a combination of quantitative NMR and LC‐MS.

During the first visit, body mass and height were measured to calculate body mass index. Body composition was then assessed using dual‐energy X‐ray absorptiometry (Discovery Series W; Hologic, Mississauga, Canada). Finally, blood pressure and peak oxygen consumption (${\dot{V}}_{O_{2}\mathrm{peak}}$) were measured. Following these baseline assessments, participants performed a familiarisation trial to become accustomed to the exercise protocol, experimental procedures and equipment used during the subsequent sessions.

Participants completed four separate experimental sessions involving HIIE under the following conditions: (1) normoxic exercise/normoxic recovery (N_Ex_‐N_Rec_); (2) hypoxic exercise/hypoxic recovery (H_Ex_‐H_Rec_); (3) normoxic exercise/hypoxic recovery (N_Ex_‐H_Rec_); and (4) hypoxic exercise/normoxic recovery (H_Ex_‐N_Rec_). The study protocol is shown in Figure 1. Before each session, participants were instructed to avoid vigorous exercise for 24 h and abstain from caffeine for 12 h. They were also asked to consume their last meal 2–3 h before arrival at the laboratory and to replicate this meal as closely as possible before each trial. A pragmatic research design was adopted, based on perceptually regulated exercise intensity. Variations in mechanical load between conditions were anticipated a priori, and findings were interpreted accordingly.

### 2.4 Baseline and familiarisation session

Detailed procedures for this session have been described previously (Soo et al., 2025). Briefly, at baseline, participants underwent an incremental cycling test to exhaustion on an electromagnetically braked cycle ergometer (Lode Corival, Groningen, The Netherlands) to assess peak oxygen consumption (${\dot{V}}_{O_{2}\mathrm{peak}}$), heart rate (HR) and associated peak power output (*W*_peak_). Following the incremental exercise, participants rested for ∼30 min before completing a familiarisation for the actual exercise intervention. This included a ramped cycling test on an electronically braked cycle ergometer (Velotron, RacerMate, Seattle, WA, USA) to help participants identify and become accustomed to intensities corresponding to a RPE of 16 (‘hard’ to ‘very hard’) and RPE of 10 (‘very light’ to ‘light’) on the Borg 6–20 scale. Finally, they performed 2 × 1‐min high‐intensity efforts at an RPE of 16, each followed by 4 min of recovery at an RPE of 10. Exercise intensity (i.e., workload) was blinded to participants throughout all sessions.

### 2.5 Experimental trials

At each experimental session, participants arrived at the lab and were instructed to rest in a seated position for 5–10 min before a venous blood sample was collected from the antecubital vein for subsequent metabolomic analysis (described below). A second venous blood sample was taken from the forearm via venepuncture immediately post‐exercise (within 10 min).

The HIIE protocol consisted of 6 × 1‐min work bouts, each interspersed with 4 min of recovery, for a total exercise duration of 30 min. Participants performed the work and recovery bouts at a perceptually regulated intensity corresponding to an RPE of 16 and 10, respectively. Cadence and gearing were self‐selected to help participants maintain the target intensity for each phase. Verbal encouragement was provided every 15 s during each 1‐min bout to ensure adherence to the prescribed effort. All testing sessions were conducted using the same Velotron cycle ergometer.

Power output was calculated and sampled at a rate of 1 Hz via an integrated algorithm within the Velotron software and subsequently exported for offline analysis. The mean power output (MPO) was determined for each 1‐min work bout, and the average MPO across all six work bouts was determined for each session. All sessions were conducted under thermo‐neutral conditions (20°C, 40% relative humidity) in an environmental chamber.

### 2.6 Hypoxia exposure

Participants wore a facemask that was connected to a portable hypoxic generator (AltiTrainer; Smtec AG, Kleinandelfingen, Switzerland) through corrugated plastic tubing during all experimental sessions. A two‐way valve attached to the corrugated tubing enabled switching between normoxic (fraction of inspired oxygen [$F_{iO_{2}}$] ∼0.21) and hypoxic air ($F_{iO_{2}}$ ∼0.14, simulating an altitude of ∼3000 m). For blinding purposes, the hypoxic generator was turned on for all experimental sessions, and the generator and two‐way valve system were positioned outside the environmental chamber (i.e., outside of the participants’ view). Additionally, oxygen saturation readings were masked from participants.

### 2.7 Exercise responses

Heart rate, and perceptual responses (difficulty breathing and limb discomfort, assessed via a modified Borg CR10 scale (Christian et al., 2014)) were recorded ∼10 s after each 1‐min bout. Peripheral oxygen saturation ($S_{pO_{2}}$, WristOx2 3150; Nonin Medical Inc., Amsterdam, the Netherlands) response was measured continuously throughout exercise. Capillary blood samples were taken from the fingertip and analysed for blood lactate levels with a lactate analyser (Lactate Plus, Nova Biomedical, Waltham, MA, USA) at baseline (before any exercise), 1 min before the start of the HIIE protocol, ∼15 s after each work bout, and ∼45 s before the start of the next 1‐min bout. The area under the curve (AUC) for $S_{pO_{2}}$ and blood lactate during HIIE was calculated using the trapezoid method from the baseline value for each participant.

### 2.8 Metabolomics analysis

Venous blood samples (6 mL) were collected in lithium heparin‐coated tubes before and after HIIE for metabolomics analyses. Samples were immediately centrifuged at 1200 *g* for 10 min, and blood plasma was transferred into microtubes for storage at −80°C until subsequent analysis. Metabolic profiling was performed on the plasma to quantify metabolites using a combination of quantitative NMR spectroscopy for the measurement of lipoproteins and small molecules. Additionally, LC‐MS was used for both targeted (i.e., branched chain amino acids) and non‐targeted (i.e., discovery) measurements of plasma metabolites, as well as for semiquantitative measurement of lipids.

### 2.9 ^1^H‐NMR spectroscopy

#### 2.9.1 ^1^H‐NMR spectroscopy sample preparation

On the day of analysis, plasma samples were thawed at room temperature for 1 h prior to preparation and centrifuged at 13,000 *g* for 10 min at 4°C. Samples were prepared in 3‐mm outer diameter SampleJet™ NMR tubes, following the procedures for In Vitro Diagnostics Research (IVDr) methodologies. Specifically, 90 µL of plasma was mixed with 90 µL of phosphate buffer (75 mM Na_2_HPO_4_, 2 mM NaN_3_, 4.6 mM sodium trimethylsilyl propionate‐[2,2,3,3‐^2^H_4_] (TSP) in D_2_0, pH 7.4 ± 0.1). Samples were sealed with a polyoxymethylene or polyacetal ball added to the cap, then manually shaken for several seconds, and stored at 5°C inside the SampleJet automatic sample changer until measurement.

#### 2.9.2 ^1^H‐NMR spectroscopy data acquisition

NMR spectroscopic analyses were performed on a 600 MHz Bruker Avance III HD spectrometer (Bruker Daltonics, Billerica, MA, USA) equipped with a 5‐mm BBI probe and fitted with the Bruker SampleJet robot. A full quantitative calibration was completed before analysis using previously published methods (Dona et al., 2014). Briefly, a sample containing 99.8% methanol‐d4 was used to validate the internal sample temperature at 310 ± 0.1 K. A 2 mM sucrose sample in H_2_O/D_2_O 9:1 was used to check for sufficient shimming quality (linewidth), solvent suppression and signal‐to‐noise performance. Lastly, quantitative referencing of the 1D‐NMR spectra was achieved using the (PUlse Length‐based CONcentration) PULCON method in combination with an external quantification reference sample (Bruker's QuantRefC – 5 mm sample) to add an artificial signal representing a predefined fixed proton concentration into the spectrum (Wider & Dreier, 2006). For each sample, two experiments were completed in automation mode (total acquisition time: 23 min), a standard 1D experiment with solvent pre‐saturation (128 scans, 98K data points, spectral width of 30 ppm) and a JEDI‐PGPE experiment (256 scans, 98K data points, spectral width of 30 ppm (Nitschke et al., 2022). Lipoprotein parameters for each sample were generated using the Bruker IVDr Lipoprotein Subclass Analysis (B.I.LISA) method (Jiménez et al., 2018). Although the lipoprotein data describe chemical components of cholesterol, free cholesterol, phospholipids, triglycerides, apolipoproteins A1/A2/B100 and the B100/A1 ratio across major lipoprotein classes (high‐density, intermediate‐density, low‐density and very‐low density lipoprotein), this study focused exclusively on very‐low‐density lipoprotein triglycerides (VLDL‐TAG). The VLDL subfractions into five density classes (VLDL1‐5) and triglyceride concentrations were obtained for each density class as well as the total VLDL‐TAG concentration, which represents the pooled triglyceride content across all VLDL density classes.

### 2.10 LC‐MS lipid analysis

Plasma lipids were analysed by liquid chromatography–triple quadrupole mass spectrometry (LC‐QQQ‐MS) as previously described (Ryan et al., 2023). Briefly, 10 µL of plasma samples were vortexed mixed with 90 µL of propan‐2‐ol containing stable isotope labelled (SIL) internal standards (Lipidyzer Internal Standards Kit from Sciex (Framingham, MA, USA), SPLASH LipidoMIX, Lyso PI 17:1, Lyso PG 17:1, and Lyso PS 17:1 (Avanti Polar Lipids, Alabaster, AL, USA)). Samples were centrifuged at 14000 *g* for 10 min with the resulting supernatant (∼70 µL) was transferred to a 96‐well plate for LC‐QQQ‐MS analysis.

The reverse‐phase chromatographic separation was performed on a SCIEX ExionLC. Separation was performed using a Waters Acquity BEH C18 1.7 µm, 2.1 × 100 mm column (Waters Corp., Milford, MA, USA) at 60°C. Mobile phase A was water/acetonitrile/propan‐2‐ol (50/30/20, v/v/v) containing 10 mM ammonium acetate, and mobile phase B was propan‐2‐ol/acetonitrile/water (90/9/1, v/v/v) containing 10 mM ammonium acetate. The injection volume was 5 µL, and samples were kept in the autosampler at 10°C. Mass detection was performed using a SCIEX QTRAP 6500+ with electrospray ionisation operated in low mass mode with polarity switching. Time‐scheduled multiple reaction monitoring (MRM) was used for the data acquisition of 1231 transitions (including 1163 lipid species and 68 internal standards). Data acquisitions were performed using SCIEX Analyst v1.7.1.

Although quantitative data for a broad range of lipid classes and individual lipid species were generated, only free fatty acids (FFA) and triglycerides (TAG) are reported in this study. A total of 19 and 443 FFAs and TAGs were detected and classified according to their degree of saturation (saturated, monounsaturated and polyunsaturated); total FFA and TAG concentration were calculated as the sum of all quantified FFA and TAG species, respectively.

### 2.11 LC‐MS amino acid analysis

Plasma amines and amino acid were analysed using a previously reported LC‐QQQ‐MS method (Gray et al., 2021). In brief, 10 µL of each plasma sample was thawed and diluted 1:1 with water followed by the addition of 20 µL of stable isotope‐labelled internal standards in water. Protein precipitation was facilitated by adding 90 µL of methanol, then samples were mixed and centrifuged at 200 *g* for 10 min at 4°C. Next, 10 µL of the supernatant was transferred into a 700 µL 96‐well plate for derivatisation with 6‐aminoquinolyl‐*N*‐hydroxysuccinimidyl carbamate (AQC). The subsequent derivatised samples were diluted 1:4 (v/v) with water prior to LC‐MS analysis.

Separation of analytes was performed on an Acquity UPLC HSS T3 1.8 µm 2.1 × 150 mm column (Waters Corp.). Eluent A consisted of 2 mM ammonium acetate in water and eluent B consisted of 2 mM ammonium acetate acetonitrile/water 95/5 (v/v). The flow rate was 0.6 mL/min and column temperature was maintained at 45°C. The autosampler compartment was cooled to 4°C and 2 µL injection volume was performed using full‐loop injection mode. Mass detection was performed using positive electrospray ionisation on quadrupole time‐of‐flight (QToF) mass spectrometers (Bruker Impact II). QToF instruments were operated to collect full scan MS data in with mass range of 30–1000 *m*/*z* and MS/MS fragmentation spectra in the same analytical run using a data independent modality offered by the Bruker broadband collision‐induced dissociation (bbCID) function.

### 2.12 Data pre‐processing

#### 2.12.1 NMR spectroscopy data processing and analysis

Data were processed in automation using Bruker Topspin 3.6.3 and ICON NMR automation to achieve phasing and baseline correction. In addition to the information extracted from the full spectral data from the standard 1D experiments, lipoprotein parameters for each sample were generated using the Bruker IVDr Lipoprotein Subclass Analysis (B.I.‐LISA) method whereby the –(CH_2_)*n* at δ = 1.25 and –CH3 at δ = 0.80 peaks of the 1D spectrum after normalisation to the Bruker QuantRef manager within Topspin were quantified using a PLS‐2 regression model. In addition, low molecular weight metabolite concentrations were obtained from the Bruker IVDr Quantification in plasma/serum B.I.Quant‐PS. All 1D NMR spectra with solvent suppression and JEDI‐PGPE spectra were normalised using ERETIC 36.

#### 2.12.2 Lipid data processing

Raw data were integrated in Skyline v21.2 (Seattle, WA, USA) (Adams et al., 2020), and concentrations of the detected lipids were calculated from the analyte‐to‐internal standard ratios. The resulting data matrix was pre‐processed using R v4.4.1 in RStudio v1.4.1 (Boston, MA, USA), where samples/features were filtered out if more than 50% of the values were missing. Subsequently, the remaining samples with <50% missing values were imputed by the minimum value/2 and a final filtering out of features that had a >30% relative standard deviation (RSD) across the replicate quality control plasma samples. The signal drift was controlled using the Random Forrest method from the *statTarget* package in the R software environment (Luan et al., 2018).

#### 2.12.3 Amino acid analysis

Obtained raw data files were processed for peak integration and quantification using the Target Analysis for Screening Quantification (TASQ; v2.2) software (Bruker Daltonics, Bremen, Germany), where calibration curves were linearly fitted with a weighting factor of 1/*x*.

### 2.13 Statistical analysis

For the statistical analysis of mechanical output (averaged MPO) and physiological responses ($S_{pO_{2}}$ and blood lactate AUC), a linear mixed method model was used, which included condition (N_Ex_‐N_Rec_, H_Ex_‐H_Rec_, N_Ex_‐H_Rec_ and H_Ex_‐N_Rec_) as fixed effects and participants as random effects. Similarly, for the statistical analysis of all metabolites associated with pyruvate metabolism and lipid oxidation, a linear mixed method model was used where condition (N_Ex_‐N_Rec_, H_Ex_‐H_Rec_, N_Ex_‐H_Rec_ and H_Ex_‐N_Rec_) and time (pre and post) were included as fixed effects, and participants included as random effects. The primary hypothesis of interest was the condition‐by‐time interaction, with main effects of condition and time examined with pairwise comparisons. For these analyses, effect size estimates, Hedges’ *g*, were also calculated, and interpreted as small (*g* = 0.2), moderate (*g* = 0.5) or large (*g* = 0.8). Variables were deemed significant when *P*‐values ≤ 0.05. Estimation of the false discovery rate due to multiple comparisons (for metabolites) was performed using the Benjamini and Hochberg method (Benjamini & Hochberg, 1995), and *P*‐values ≤0.05 with a *q*‐value (false discovery rate‐adjusted *P*‐values) ≤0.05 were considered statistically significant. Statistical analyses of the metabolomic dataset were performed using R (v4.5.0).

## 3 RESULTS

### 3.1 Exercise performance and physiological responses during HIIE

Averaged MPO, was lower in H_Ex_‐H_Rec_ compared to N_Ex_‐N_Rec_, N_Ex_‐H_Rec_, and H_Ex_‐N_Rec_ (all *P* ≤ 0.009; *g *= 0.37–0.59; Figure 2a), with no differences (all *P* ≥ 0.11; *g *= 0.02–0.21) between the other conditions. Blood lactate response did not differ between conditions (*P *= 0.41; *g *= 0.02–0.19; Figure 2b). The $S_{pO_{2}}$ response was lower in H_Ex_‐H_Rec_ compared to N_Ex_‐N_Rec_, N_Ex_‐H_Rec_ and H_Ex_‐N_Rec_ (all *P *< 0.001; *g *= 1.88–5.83; Figure 2c). Additionally, $S_{pO_{2}}$ in N_Ex_‐H_Rec_ and H_Ex_‐N_Rec_ was lower compared to N_Ex_‐N_Rec_ (both *P *< 0.001; *g *= 0.55 and 4.18, respectively).

**FIGURE 2:**
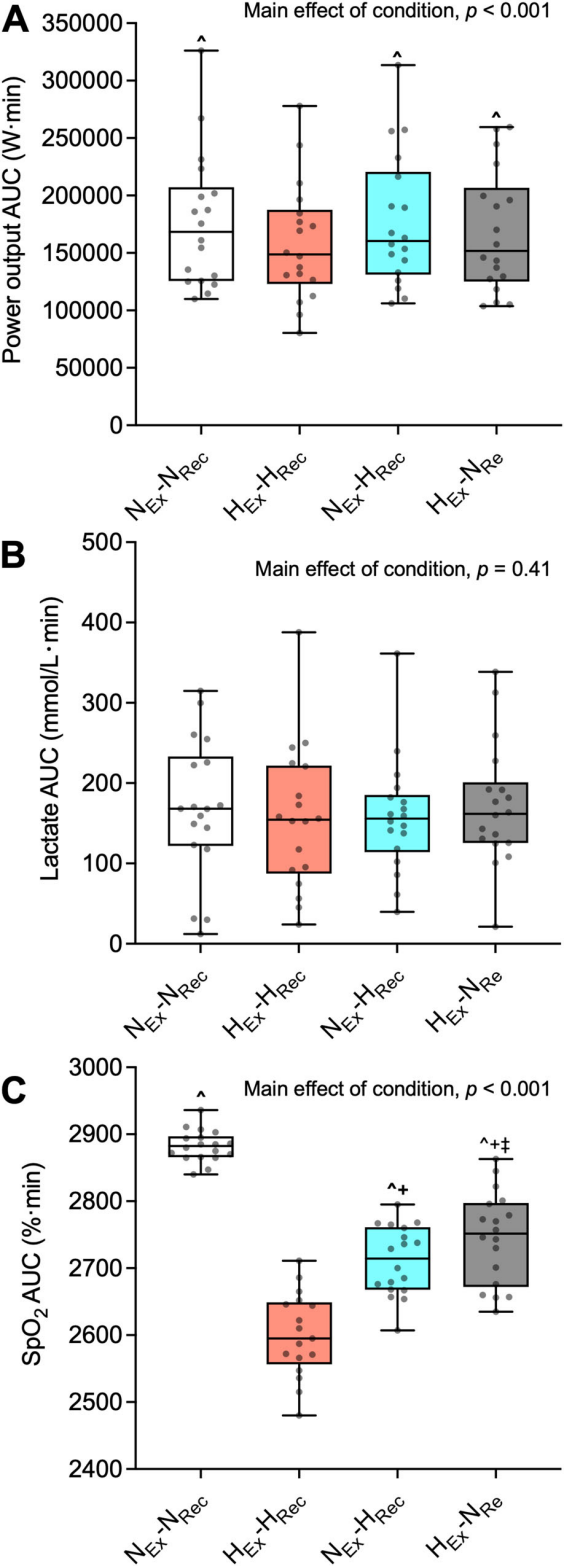
Mean power output under the curve (across the six 1‐min work bouts) (a), lactate area under the curve (measured at baseline, ∼45 s before and ∼15 s after each work bout) (b), and peripheral oxygen saturation area under the curve ($S_{pO_{2}}$, measured continuously) (c) during the high intensity interval exercise under normoxic exercise/normoxic recovery (N_Ex_‐N_Rec_), hypoxic exercise/hypoxic recovery (H_Ex_‐H_Rec_), normoxic exercise/hypoxic recovery (N_Ex_‐H_Rec_) and hypoxic exercise/hypoxic recovery (H_Ex_‐N_Rec_). *n* = 18. *Significant difference from H_Ex_‐H_Rec_; ^+^significant difference from N_Ex_‐N_Rec_; ^‡^significant difference from N_Ex_‐H_Rec_; *P *< 0.05.

### 3.2 Pyruvate metabolism

Irrespective of conditions, plasma concentrations of pyruvate (*P *< 0.001; *q *< 0.001; *g *= 1.58), lactate (*P *< 0.001; *q *< 0.001; *g *= 1.62) and citrate (*P *< 0.001; *q *< 0.001; *g *= 1.41) significantly increased following HIIE (Figure 3b, c, d). The lactate‐to‐pyruvate ratio also increased across all conditions post‐HIIE (*P *< 0.001; *g *= 1.17; Figure 4). Irrespective of conditions, plasma concentrations of valine (main effect of time, *P *= 0.02; *q *= 0.04; *g *= 0.37; Figure 3i), leucine (*P *= 0.002; *q *= 0.005; *g *= 0.51; Figure 3g), isoleucine (*P *= 0.001; *q *= 0.004; *g *= 0.52; Figure 3h) and aspartate (*P *= 0.01; *q *= 0.02; *g *= 0.36; Figure 3f) decreased following HIIE. While alanine concentration increased following HIIE across conditions (main effect of time, *P *< 0.001; *q *= 0.003; *g *= 0.62; Figure 3e), there were no changes in glutamate (main effect of time, *P *= 0.25; *q *= 0.36; *g *= 0.12; Figure 3k) and glutamine concentrations (*P *= 0.37; *q *= 0.47; *g *= 0.12; Figure 3l).

**FIGURE 3:**
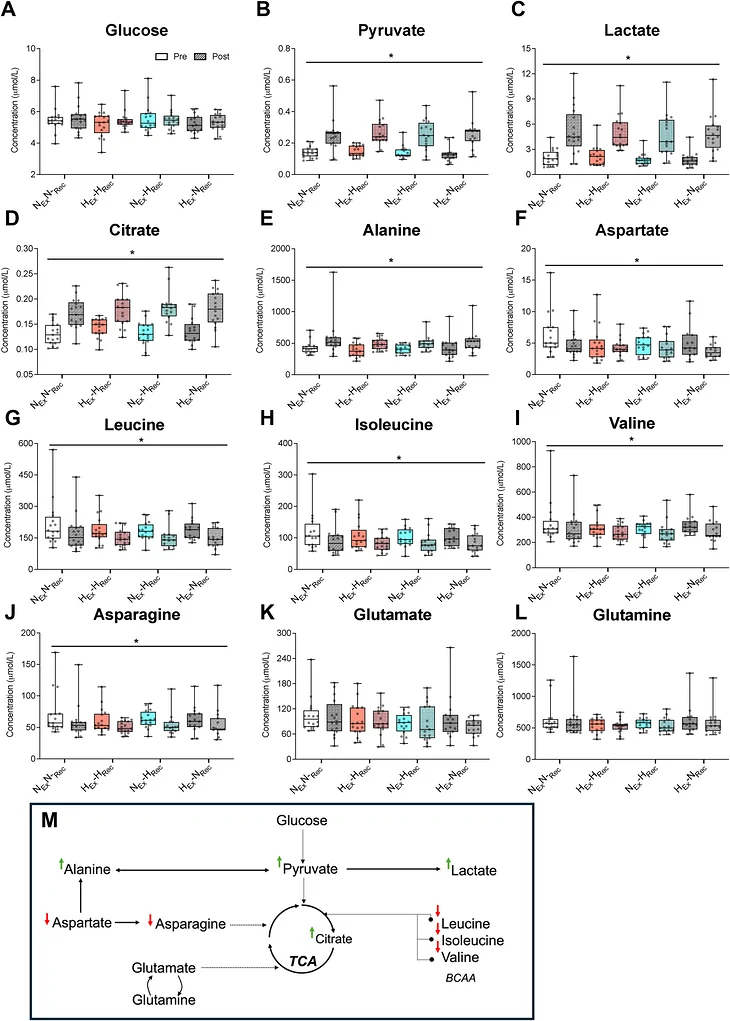
Pyruvate metabolism. (a–l) Changes in plasma concentration of glucose, pyruvate, lactate, citrate, alanine, aspartate, leucine, isoleucine, valine, asparagine, glutamate and glutamine before and after the high intensity interval exercise under normoxic exercise/normoxic recovery (N_Ex_‐N_Rec_), hypoxic exercise/hypoxic recovery (H_Ex_‐H_Rec_), normoxic exercise/hypoxic recovery (N_Ex_‐H_Rec_) and hypoxic exercise/hypoxic recovery (H_Ex_‐N_Rec_). *n* = 18. *Time effect: *P *< 0.05 and *q* < 0.05. (m) Schematic representation of identified metabolites of pyruvate metabolism. Green and red arrow indicates increased and decreased metabolites post‐exercise, respectively, across all conditions.

**FIGURE 4:**
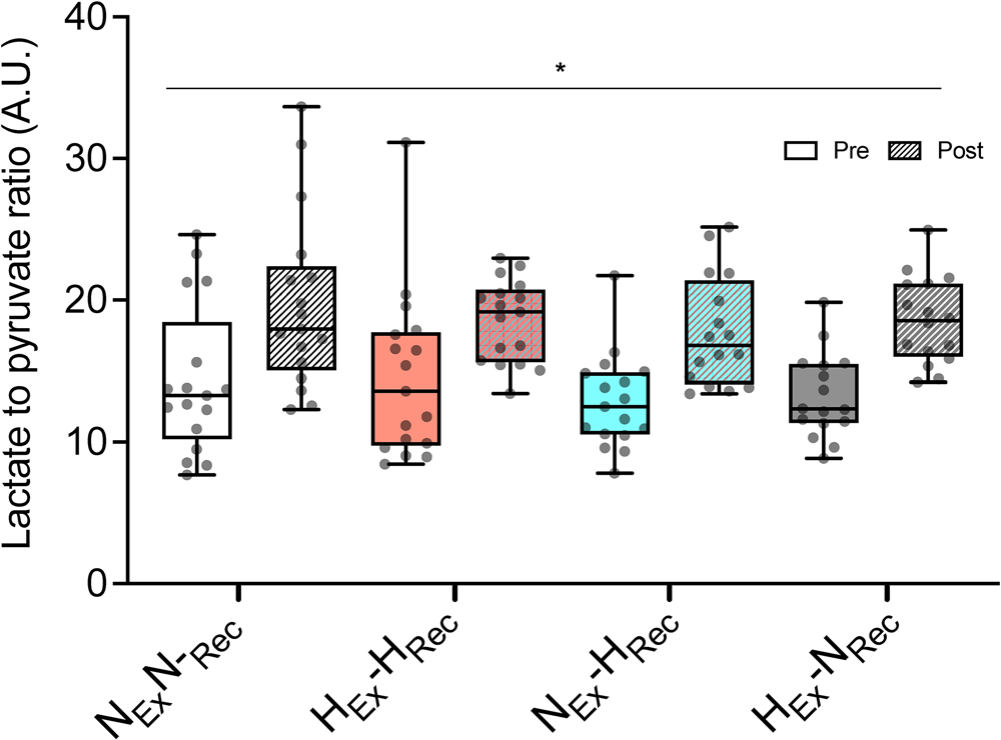
Lactate‐to‐pyruvate ratio measured before and after the high intensity interval exercise under normoxic exercise/normoxic recovery (N_Ex_‐N_Rec_), hypoxic exercise/hypoxic recovery (H_Ex_‐H_Rec_), normoxic exercise/hypoxic recovery (N_Ex_‐H_Rec_) and hypoxic exercise/hypoxic recovery (H_Ex_‐N_Rec_). *n* = 18. *Time effect: *P *< 0.05.

### 3.3 Lipid metabolism

Within the very low‐density lipoprotein (VLDL) subfraction (Figure 5a), total concentration of VLDL‐TAG was not altered by condition (*P *= 0.33; *q *= 0.90) or time (*P *= 0.51; *q *= 0.40). However, an increase in plasma concentration of VLDL4‐TAG (*P *= 0.02; *g *= 0.19; Table 1) was observed, with no differences between conditions (*P *= 0.76). Additionally, irrespective of condition, plasma concentration of VLDL5‐TAG (*P *= 0.001; *g *= 0.35) was reduced following HIIE.

**FIGURE 5:**
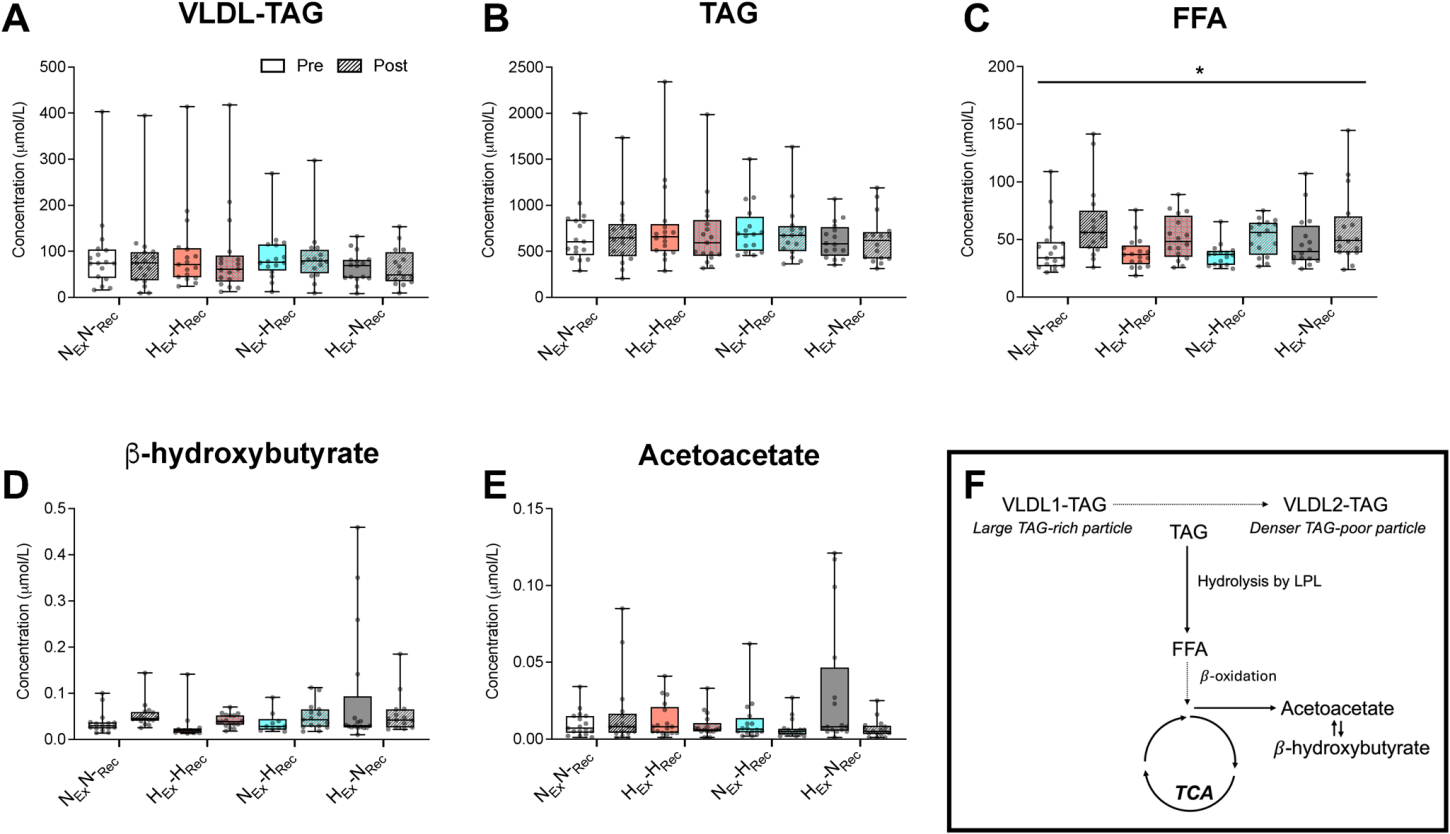
Lipid oxidation. (a–e) Changes in plasma concentration of VLDL‐TAG, TAG, FFA, β‐hydroxybutyrate and acetoacetate before and after the high intensity interval exercise under normoxic exercise/normoxic recovery (N_Ex_‐N_Rec_), hypoxic exercise/hypoxic recovery (H_Ex_‐H_Rec_), normoxic exercise/hypoxic recovery (N_Ex_‐H_Rec_) and hypoxic exercise/hypoxic recovery (H_Ex_‐N_Rec_). (f) Overview of fatty acid oxidation pathway. *n* = 18. *Time effect: *P *< 0.05 and *q* < 0.05.

**TABLE 1:** Changes in plasma very‐low density‐triglyceride, triglyceride and free fatty composition during high intensity interval exercise under normoxic exercise/normoxic recovery (N_Ex_‐N_Rec_), hypoxic exercise/hypoxic recovery (H_Ex_‐H_Rec_), normoxic exercise/hypoxic recovery (N_Ex_‐H_Rec_), and hypoxic exercise/hypoxic recovery (H_Ex_‐N_Rec_).

	Condition								*p*‐value		
	N_Ex_‐N_Rec_		H_Ex_‐H_Rec_		N_Ex_‐H_Rec_		H_Ex_‐N_Rec_		Condition	Time	Condition × Time
	Pre	Post	Pre	Post	Pre	Post	Pre	Post			
VLDL1‐TAG (µM)	57.4 (66.3)	50.6 (66.5)	62.5 (70.4)	56.6 (76.0)	51.1 (35.4)	46.7 (41.3)	36.6 (21.0)	35.3 (27.4)	0.24	0.10	0.87
VLDL2‐TAG (µM)	13.8 (10.8)	14.0 (11.2)	14.1 (11.8)	14.3 (12.2)	15.3 (11.8)	16.0 (11.8)	10.5 (6.2)	10.5 (7.2)	0.07	0.79	1.00
VLDL3‐TAG (µM)	10.9 (8.2)	12.0 (8.3)	10.8 (8.0)	12.3 (8.9)	12.0 (8.9)	13.3 (8.4)	9.3 (4.6)	9.3 (5.7)	0.10	0.05	0.93
VLDL4‐TAG (µM)	8.1 (4.3)	9.0 (4.5)	7.9 (3.7)	8.9 (4.2)	8.1 (3.7)	9.0 (3.7)	7.4 (2.9)	7.6 (3.0)	0.81	0.02	0.76
VLDL5‐TAG (µM)	2.6 (0.8)	2.3 (0.9)	2.3 (0.8)	2.1 (0.8)	2.2 (0.4)	2.1 (0.5)	2.3 (0.5)	2.1 (0.6)	0.12	0.001	0.92
**TAG**											
Saturated (µM)	29.5 (21.2)	20.6 (15.9)	20.0 (15.5)	21.8 (15.8)	31.8 (18.8)	24.0 (18.3)	25.9 (14.5)	22.8 (20.3)	0.10	0.004	0.60
Monounsaturated (µM)	74.6 (53.1)	63.3 (45.8)	76.2 (60.6)	65.0 (49.9)	78.6 (41.4)	70.8 (47.4)	63.7 (28.1)	63.0 (47.4)	0.77	0.04	0.74
Polyunsaturated (µM)	603.9 (327.5)	592.7 (291.5)	675.1 (405.0)	610.6 (342.6)	629.9 (232.0)	611.7 (249.8)	530.0 (159.2)	544.1 (202.6)	0.46	0.33	0.69
**FFA**											
Saturated (µM)	8.6 (5.2)	13.7 (8.5)	7.5 (3.3)	10.2 (4.1)	7.7 (3.7)	11.7 (4.8)	10.2 (6.3)	12.5 (7.7)	0.15	<0.001	0.65
Monounsaturated (µM)	24.5 (12.2)	35.1 (16.5)	22.2 (7.0)	29.5 (10.4)	21.4 (4.9)	29.2 (7.7)	27.2 (11.6)	33.2 (15.8)	0.10	<0.001	0.80
Polyunsaturated (µM)	9.5 (5.9)	15.2 (8.1)	8.7 (3.9)	12.4 (5.0)	7.3 (3.7)	11.7 (4.8)	10.5 (5.8)	13.7 (9.3)	0.06	<0.001	0.77

Data are the mean (standard deviation). FFA, free fatty acids; TAG, triacylglycerol; VLDL‐TAG, very‐low‐density lipoprotein triglyceride.

A total of 443 TAGs were annotated, with the total carbon count (of the three acyl chains) ranging from 40 to 60. The total plasma TAG concentration was not different following HIIE across all conditions (*P *= 0.72; *q *= 0.91; Figure 5b). However, a noticeable reduction in TAG species was observed following HIIE, irrespective of conditions, notably in saturated (*P *= 0.004) and monounsaturated (*P *= 0.04) TAG (Table 1).

A total of 19 FFAs were annotated, with the total carbon count ranging from 16 to 24. Total plasma FFA concentration increased significantly post‐HIIE (N_Ex_‐N_Rec_: +50%, *g* = 0.75; H_Ex_‐H_Rec_: +36%, *g* = 0.80; N_Ex_‐H_Rec_: +43%, *g *= 1.31; and H_Ex_‐N_Rec_: +24%, *g *= 0.40; main effect of time, *P *< 0.001; *q *= 0.001; Figure 5c), with no significant difference between conditions (*P *= 0.75; *q *= 0.72). Increases in plasma concentration of saturated, monounsaturated and polyunsaturated fatty acids were observed following HIIE in all conditions (all *P *< 0.001; Table 1). With regards to ketone metabolism, there were no significant condition × time effects detected for β‐hydroxybutyrate (*P *= 0.02, *q *= 0.19) and acetoacetate (*P *= 0.009, *q *= 0.17).

## 4 DISCUSSION

This study examined the effects of intermittent and continuous hypoxia during perceptually regulated HIIE on the underlying plasma metabolome. The key findings were: (1) continuous hypoxia decreased MPO; (2) despite varying decreases in $S_{pO_{2}}$ across conditions (i.e., greatest in H_Ex_‐H_Rec_, followed by N_Ex_‐H_Rec_, H_Ex_‐N_Rec_ and N_Ex_‐N_Rec_), lactate concentrations measured during and after HIIE were not different between conditions; (3) lactate‐to‐pyruvate ratios increased consistently across all conditions; (4) modest changes in amino acids associated with pyruvate metabolism (specifically alanine and glutamine) indicate limited energetic contribution from amino acids during the HIIE that was not affected by hypoxia; and (5) increased plasma FFA concentrations indicate rates of lipolysis in extra‐muscular cells and tissues exceeded skeletal muscle FFA uptake. Taken together, these findings suggest that (1) the addition of continuous hypoxia may compensate for the significant decrease in mechanical output, resulting in the same metabolic response as in normoxia; and (2) despite similar mechanical output the addition of intermittent hypoxia did not enhance the metabolic response beyond that of perceptually regulated HIIE in normoxia.

### 4.1 Pyruvate metabolism

The rate of anaerobic glycolysis during HIIE is influenced by both absolute exercise intensity and oxygen availability, with an increased rate of anaerobic glycolysis expected with reduced oxygen availability (McLellan et al., 1990; Morales‐Alamo et al., 2012). For these reasons, lactate is commonly measured as an indicator of pyruvate production via the glycolytic pathway exceeding the rate of utilisation by the mitochondria and/or a reduced cytosolic redox state (i.e., increased NADH:NAD^+^ ratio) leading to pyruvate being reduced to lactate. Under the current conditions, hypoxia caused decreases in $S_{pO_{2}}$, with the greatest reduction in H_Ex_‐H_Rec_, followed by N_Ex_‐H_Rec_ and H_Ex_‐N_Rec_. The modest decrease in $S_{pO_{2}}$ observed during intermittent hypoxia (N_Ex_‐H_Rec_ and H_Ex_‐N_Rec_) likely mitigated reductions in mechanical output. Despite these differences in oxygen saturation and exercise performance across conditions, lactate concentrations measured during and after HIIE did not differ and were remarkably consistent among participants who were not habitually exposed to HIIE or hypoxic training. Accordingly, the lactate‐to‐pyruvate ratio increased across all HIIE conditions, but was not further elevated by hypoxia. The lack of higher lactate concentrations (and the lactate to pyruvate ratio) following N_Ex_‐H_Rec_ and H_Ex_‐N_Rec_ compared to normoxia is unclear, but this could be due to the glycolytic rate approaching near maximal capacity during the high intensity exercise protocol employed thereby limiting any further effect of reduced O_2_ availability.

In addition to pyruvate being reduced to lactate, its accumulation also results in increased transamination to alanine (Bowtell & Bruce, 2002). This increase in pyruvate is likely due to an increase in glycolytic and glycogenolytic activity, although the origin (and contribution) is unclear. Although muscle glycogen concentrations were not measured, the concurrent increase in lactate and alanine likely indicate an increase in muscle glycogen utilisation during high‐intensity exercise (Hermansen & Vaage, 1977; Medbø, 1993). The transamination of pyruvate to alanine is regulated by alanine aminotransferase, with glutamate serving as the amino group donor (glutamate + pyruvate ⇌ alanine + 2‐oxoglutarate + NADH). This reaction is an anaplerotic metabolic reaction that facilitates replenishment of TCA cycle intermediates as the TCA flux increases. Supporting this, the observed reduction in plasma aspartate concentrations indicates increased activity of anaplerotic pathways (purine nucleotide cycle), which replenishes fumarate and adenosine monophosphate to support the TCA cycle.

Theoretically, the increase in alanine concentration (and associated aminotransferase activity) should deplete the concentration of glutamate due to increased competition for transamination of pyruvate to produce alanine, and/or for glutamine production (Babij et al., 1983). However, plasma concentrations of glutamate and glutamine were not significantly altered following HIIE in any condition. This may indicate limited uptake and utilisation of glutamate by skeletal muscle during exercise. However, the transamination of the branched chain amino acids, including leucine, isoleucine and valine, may also act as an important source of skeletal muscle glutamate (Rutten et al., 2005). Indeed, plasma concentrations of leucine, isoleucine and valine decreased across all conditions, indicating increased uptake and oxidation. It is important to highlight that the skeletal muscle is the primary site of glutamate synthesis (Bergström et al., 1974). Consequently, the skeletal muscle glutamate concentration far exceeds that in the plasma (Bergström et al., 1974; Marliss et al., 1971). Therefore, without the application of isotopic tracers, measurements of plasma glutamate concentration per se (as in this study) may not fully reflect glutamate metabolism. Nonetheless, the overall data indicate an increase in TCA cycle flux towards energy provision during HIIE, as further supported by the amino acid oxidation and anaplerotic reactions for the replenishments of TCA cycle intermediates. However, the TCA cycle flux and anaplerotic reactions were likely not altered by the addition of continuous or intermittent hypoxia.

### 4.2 Lipid metabolism

A significant increase in the concentration of plasma FFA was observed across all classes (saturated, monounsaturated and polyunsaturated) following HIIE, irrespective of conditions. In this regard, plasma FFA that are oxidised by the skeletal muscle can be derived from both the plasma non‐esterified fatty acid pool (i.e., lipolysis of TAG stores in the adipose tissue) and from the VLDL‐TAG. While a noticeable decrease in saturated and monounsaturated triglycerides was observed, overall plasma triglyceride concentrations were largely unaltered by either hypoxia or HIIE. This was likely attributed to the high abundance of polyunsaturated TAG, which remained unchanged with HIIE and hypoxia. Additionally, the VLDL subfraction demonstrated a modest decrease in VLDL5‐TAG and increases in VLDL‐4 TAG concentrations, yet overall VLDL‐TAG concentrations were unaltered by HIIE or hypoxia. These results largely reflect well‐established findings (Brooks, 1997; Brooks & Mercier, 1994) indicating that energy contribution from lipid oxidation decreases with increasing intensities. Consequently, plasma TAG and VLDL‐TAG are unlikely to serve as a major energy source during HIIE in hypoxia.

Despite only modest changes in plasma triglyceride and VLDL‐TAG concentration, an increase in the plasma FFA concentration was observed following HIIE in all conditions. This is perhaps unsurprising given that high intensity exertion (Kindermann et al., 1982) and hypoxia (Kon et al., 2015) are known to promote the release of adrenaline and noradrenaline, which stimulates lipolysis and the release of non‐esterified fatty acid into the circulation (activation of lipolysis is largely influenced by β‐adrenegic activation). While most studies typically report plasma FFA as a single entity, in‐depth lipidomic profiling enables a detailed analysis of individual FFA species that differ in chain length and saturation. Our results show that both saturated and unsaturated free fatty acids increased following HIIE and hypoxia, although the increase in plasma FFA concentration was largely driven by increases in mono‐ and polyunsaturated fatty acids (Table 1). These findings are supported by previous studies showing increased mobilisation of unsaturated compared to saturated fatty acids (Mougios et al., 1995, 1998). This could be due to the differing rates of fatty acid oxidation, with unsaturated fatty acids being oxidised more rapidly compared to saturated fatty acids (DeLany et al., 2000). That said, the increase in plasma FFA concentration in all conditions may indicate either increased mobilisation of FFA (i.e., that exceeds uptake and oxidation of FFA) or a suppression of FFA uptake (into the skeletal muscle) and oxidation, which cannot be differentiated in the current study. In this instance, studies have shown lipid oxidation contribution increases during high‐intensity cycling bouts (Stepto et al., 2001; Zagatto et al., 2022). That said, the lack of significant increases in ketone metabolites (β‐hydroxybutyrate and acetoacetate) – which would occur when acetyl‐CoA production via β‐oxidation exceeds the TCA cycle capacity – suggests decreased FFA utilisation. Given the well‐established metabolic shift during high‐intensity exercise and hypoxia towards glycolysis and increased reliance on glucose and glycogen (and down‐regulation of free fatty acid oxidation) (Brooks & Mercier, 1994; Randle et al., 1964), it is more likely that the elevated FFA concentrations primarily reflect increased lipolysis combined with a modest (or suppression) reliance on FFA oxidation for energy provision.

### 4.3 Limitations

Diet was not controlled in this study, although participants were instructed to replicate their dietary intake (for their last meal) as closely as possible. Furthermore, each experimental session was conducted at similar times of the day (±2 h) to minimise the effect of diet or diurnal variation in metabolism. Nonetheless, fluctuations in metabolites such as FFAs may still have occurred due to differences in dietary fatty acid intake (Bradley et al., 2008). Additionally, the predominance of male participants (13 out of 18) limits the generalisability of the findings. Further studies should examine if sex differences or menstrual cycle phases influence the metabolome following HIIE in hypoxia.

### 4.4 Conclusion

HIIE consistently increased plasma FFA concentrations, lactate:pyruvate ratio and intermediates closely associated with pyruvate (i.e., lactate, alanine, citrate). However, exposure to hypoxia, either continuously or intermittently, did not differentially affect metabolite concentrations associated with the major energetic pathways. The similar metabolic response following HIIE in continuous hypoxia relative to normoxia is likely due to the significant decrease in total mechanical output. Conversely, despite the maintenance of mechanical output, intermittent hypoxia (N_Ex_‐H_Rec_ and H_Ex_‐N_Rec_) did not enhance the metabolic response beyond that of normoxia likely due to the metabolic rate approaching maximal capacity. Intermittent hypoxia implemented either during the work or recovery phases of HIIE attenuated decreases in oxygen saturation and exercise performance seen with continuous hypoxia. Importantly, despite this attenuation and maintenance of exercise intensity, intermittent hypoxia (applied during either work or recovery) did not enhance the major energetic pathways.

## AUTHOR CONTRIBUTIONS

Jacky Soo, Paul Goods, Olivier Girard, Louise Deldicque, Nathan Lawler and Timothy Fairchild contributed to the conception and design of the study. Jacky Soo collected the data. Jacky Soo, Maartje Cox, Monique Ryan, Philipp Nitschke, and Nathan Lawler were responsible for the analysis and interpretation of data. Jacky Soo wrote the first draft of the manuscript. All authors have reviewed the manuscript and provided critical feedback. All authors have approved the final version of the manuscript and agree to be accountable for all aspects of the work in ensuring that questions related to the accuracy or integrity of any part of the work are appropriately investigated and resolved. All persons designated as authors qualify for authorship, and all those who qualify for authorship are listed.

## CONFLICT OF INTEREST

None declared.

## FUNDING INFORMATION

None.

## GENERATIVE AI STATEMENT

The authors declare that no artificial intelligence tools, including large language models (LLMs), were used in the drafting or revision of this manuscript.

## Data Availability

The data that support the findings of this study are available from the corresponding authors upon reasonable request.
